# PrPSc with Seeding Activity Extensively Overlaps with Proteinase-Resistant PrPSc Rather than Infectious PrPSc

**DOI:** 10.3390/pathogens9030241

**Published:** 2020-03-24

**Authors:** Yoshifumi Iwamaru, Yuichi Matsuura, Kohtaro Miyazawa

**Affiliations:** Viral Ecology Unit, Division of Viral Disease and Epidemiology, National Institute of Animal Health (NIAH), National Agriculture and Food Research Organization (NARO), Tsukuba, Ibaraki 305-0856, Japan; zrxmatsu@affrc.go.jp (Y.M.); miyazawak@affrc.go.jp (K.M.)

**Keywords:** amyloid, bovine spongiform encephalopathy, hamster, infectivity, kinetics, mouse model, prion, real-time quaking induced conversion (RT-QuIC), scrapie, seeding activity

## Abstract

The disease-associated prion protein (PrPSc) has the ability to seed the conformational conversion of normal prion proteins into the amyloid fibril form. This prion seeding activity can be measured using an in vitro amplification assay termed real-time quaking-induced conversion (RT-QuIC). There is a strong correlation between RT-QuIC positivity and prion infection; however, the relationship between seeding activity and infectivity remains elusive. In this study, we used endpoint dilution RT-QuIC on the brain homogenates from wild-type mice with mouse-adopted bovine spongiform encephalopathy (mBSE) at defined intervals during the incubation period and evaluated the temporal relationship among prion seeding dose, levels of proteinase-resistant PrPSc (PrPres), and infectious titer. We found that the infectious titer reached a plateau by 100 days postinfection, whereas seeding dose and PrPres levels were continuously elevated. Our calculation showed that the doubling time (*dt*) for seeding dose from 40 to 100 days postinoculation was closer to the *dt* for PrPres levels than to the *dt* for prion titer. Although an uncoupling of seeding doses and PrPres levels was observed at end-stage disease in this model, our findings suggest that there is substantial but not complete overlap between PrPSc with seeding activity and PrPres rather than infectious PrPSc.

## 1. Introduction

Prion diseases are lethal neurodegenerative disorders that can affect both humans and animals. The pathogenesis of prion diseases requires the conversion of normal cellular prion protein (PrPC) to its disease-associated isoform (PrPSc) and is accompanied by its accumulation in the brains of affected organisms. [1]. The presence of PrPSc correlates with infectivity [2], and hence, it is believed to be the main agent of prion diseases. PrPSc exhibits partial proteolytic resistance against proteinase K (PK), and proteinase-resistant PrPSc (PrPres) has been considered a surrogate marker for prion infection.

Several studies have shown that prion infectivity does not always correlate well with PrPres levels [3]. In previous temporal correlative studies on infectious titers and PrPres levels in prion-infected animals, a continuous increase in PrPres levels was observed even after the infectious titer reached a plateau [4,5,6,7]. In addition, high levels of infectivity with undetectable or extremely low PrPres were observed in transgenic mice expressing murine PrP P101L (Tg 101LL) with Gerstmann–Sträussler–Scheinker syndrome [8,9] or 263K scrapie [10].

Real-time quaking-induced conversion (RT-QuIC) is one of the novel in vitro amplification assays that was developed based on the prion-seeded conversion of recombinant prion proteins (recPrP) to the amyloid fibrillar form by shaking [11,12] rather than the sonication used in its original assay [13]. There is a strong correlation between RT-QuIC positivity and prion infection. The RT-QuIC assay has been shown to detect prion seeding activity in tissues and body fluids of animals and humans with prion diseases, with sensitivities higher than those of animal bioassays [11]. Thus, RT-QuIC can act as a possible antemortem test for prion disease [12], an evaluation tool for decontamination methods [14], and a risk assessment tools for iatrogenic prion transmission via tissues [15].

Considering the aforementioned discrepancies between PrPres and prion infectivity, it is important to establish the relationship between prion seeding activity and infectivity. A previous study reported a closer correlation of prion seeding activity with infectivity than with PrPres levels, as judged by the similar seeding activities in the brains of 139A or 263K scrapie-affected Tg 101LL mice containing equivalent infectious titers but discrepant PrPres levels [16]. On the other hand, it has also been reported that prion infectivity is eliminated by a disinfectant without altering seeding activity [17]. Hence, the controversial relationship between seeding activity and infectivity remains to be elucidated. Although time-course prion seeding activity in scrapie-infected hamsters has been described [18], the temporal relationship between prion seeding dose and infectious titer has not yet been determined.

In our previous temporal study on mouse-adopted classical bovine spongiform encephalopathy (mBSE) prion, we reported that the infectious titer reached a plateau at 100 days postinfection (dpi) whereas PrPres levels were continuously elevated towards the end of the incubation period, suggesting the uncoupling of prion titer and PrPres levels [19]. In this study, we attempted to determine the kinetics of prion seeding activity in the brains of these mice. We applied endpoint dilution RT-QuIC on brain homogenates to obtain a detailed measurement of seeding activities at defined intervals during the incubation period in mice and assessed the temporal relationships among seeding dose, PrPres levels, and prion titer. In addition, we investigated the alterations in seeding doses associated with the reduction of PrPres levels after high-speed centrifugation of brain homogenates.

## 2. Results

### 2.1. RT-QuIC Detection of mBSE BH-Derived Seeding Activity

**Figure 1:**
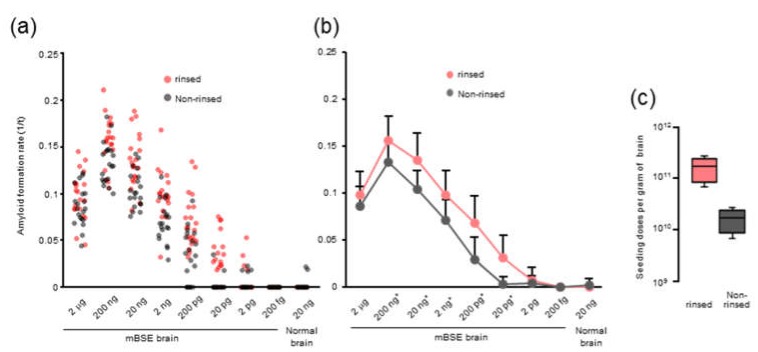
Real-time quaking-induced conversion (RT-QuIC) for the detection of prion seeding activity in a brain from a terminally ill CD-1 mouse inoculated with mouse-adapted BSE (mBSE): Syrian hamster recombinant prion protein (amino acids 90–231) was used as a substrate for the RT-QuIC reactions. The seeded brain tissue equivalents (2 µg to 200 fg from an mBSE-inoculated mouse or with 20 ng brain tissue from a normal mouse) are shown at the bottom. (**a**) Each dot represents the amyloid formation rate (AFR) measured in a 96-well plate rinsed with (pale red) or without (grey) an acetone-ethanol mixture. The 96-well plates from three lot numbers were used for the four analyses. (**b**) Line plot of Figure 1a. The mean ± SD of the AFR is displayed for each dilution. Significant differences at *p* < 0.05 (*) between AFR measured in rinsed and non-rinsed 96-well plates are indicated. (**c**) Each box represents Spearman–Käber estimates of the SD_50_ per unit per gram of brain tissue measured in a 96-well plate rinsed with (pale red) or without (grey) the acetone-ethanol mixture. Data are derived from quintuplicate wells of four experiments for each brain tissue dilution.

A previous study indicated that RT-QuIC using N-terminally truncated hamster recombinant PrP residues 90–231 (recPrP) could detect a mouse-adopted scrapie RML strain in wild-type mice [20]. To determine if prion seeding activity derived from mBSE in CD-1 mice can be detected, we performed RT-QuIC using hamster recPrP. Quintuplicate RT-QuIC reactions were seeded with serial dilutions of the brain homogenates (BHs) of terminally ill mice with mBSE (from a 10^−2^ to 10^−9^ dilution of a 10% weight per volume BH). We used 96-well plates from three lot numbers for the four analyses. In parallel, we assessed the effect of rinsing the 96-well plates with an acetone-ethanol 1/9 (v/v) mixture, as Hoover and colleagues demonstrated that the inhibitory effect of brain lipids on prion amyloid formation can be abrogated using an alcohol-based extraction technique [21]. With the mBSE strain, seeding activity was detected using RT-QuIC and hamster recPrP. The amyloid formation rate, which is represented by the inverse of the time (h) for the thioflavin T fluorescence signals to exceed the threshold for each replicate seeded with serially-diluted brain tissues, is shown in Figure 1a. The amyloid formation rate, which was measured using non-treated 96-wells plates, had a linear detection range from 200 ng (dilutions of 1 × 10^−3^) to 20 pg (dilutions of 1 × 10^−7^) (Figure 1b). The number of the positive replicates started to decrease at 200 pg seeded, and the mean ± SD of Spearman–Käber estimates of the SD_50_ per unit tissue was 10.15 ± 0.06 log SD_50_ per gram of brain (Figure 1c).

After rinsing the 96-well plates with the acetone-ethanol mixture, we observed significantly higher rates of amyloid formation from 10^−3^ to 10^−7^ dilutions than those measured using the non-treated 96-well plates. The amyloid formation rates that were measured in the acetone-ethanol mixture rinsed 96-well plates displayed a linear detection range from 200 ng (dilutions of 1 × 10^−3^) to 2 pg (dilutions of 1 × 10^−8^). In addition, the SD_50_ per unit of the same tissue was 11.15 ± 0.06 log SD_50_ per gram of brain, indicating that rinsing the 96-well plates with an acetone-ethanol mixture improved the RT-QuIC detection limit by an order of magnitude without increasing the false positive rate. Similar improvements in the RT-QuIC detection limit and amyloid formation after rinsing the 96-well plates were also observed for chronic wasting disease (CWD)-affected elk BH (Figure S2). Hence, we used 96-well plates rinsed with the acetone-ethanol mixture for subsequent RT-QUIC analyses. For mechanistic insight, we assessed the adsorption of recPrP to the 96-well plates rinsed with or without the acetone-ethanol mixture; however, bicinchoninic acid assays showed no significant difference in the surface adsorption of recPrP (data not shown). Investigations on the precise effect of organic solvents in improving prion amyloid formation might possibly provide clues regarding the mechanistic details of amyloid assembly.

### 2.2. Correlation among Seeding Activity, Infectivity, and PrPres Levels

**Figure 2:**
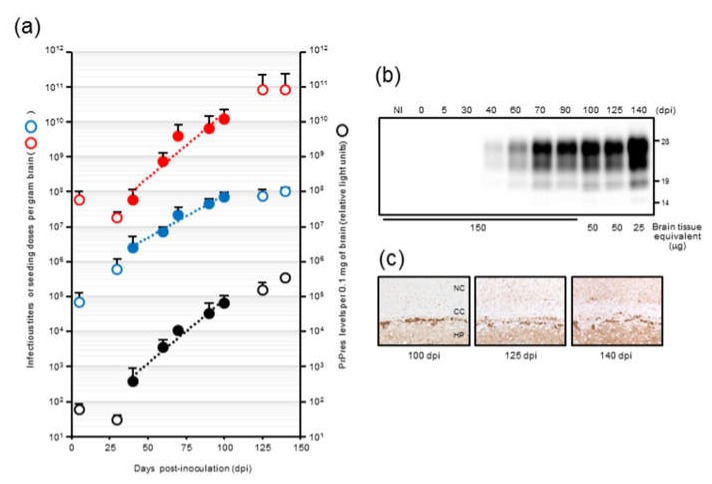
Kinetics of mouse-adopted bovine spongiform encephalopathy (mBSE) prion seeding activity, infectivity, and PrPres (proteinase-resistant disease-associated prion protein (PrPSc)) accumulation in mouse brains during disease progression: (**a**) Seeding dose (red circle), infectious titer (blue circle), and PrPres accumulation (black circle) in mouse brains are shown. The seeding doses of mBSE prion at each time point were determined using end-point dilution RT-QuIC. Each blue circle represents the mean ± SD of Spearman–Käber estimates of the SD_50_ per gram of brain and was derived from quadruplicate wells of at least three experiments. The infectious titers of mBSE prion per gram of brain at each time point were estimated using the incubation period bioassay method. The levels of PrPres in 0.1 mg brain equivalents at the same time points were measured using ELISA with the Seprion ligand derived from triplicate wells of two experiments and plotted on a log scale in arbitrary light units. The kinetics of seeding activity, infectivity, and PrPres accumulation are shown as a semilogarithmic plot. A linear relationship was observed from 40 to 100 dpi of seeding activity (red filled circle and dashed line: y = 3,541,353* e^(0.085361x) R^2^ = 0.905892, where y is the seeding dose and x is the survival days), infectivity (blue filled circle and dashed line: y = 296,647* e^(0.056066x) R^2^ = 0.970865, where y is the infectious titer and x is the survival days), and PrPres accumulation (black filled circle and dashed line: y = 19.2724* e^(0.083569x) R^2^ = 0.972250, where y is the relative light units and x is the survival days). (**b**) Western blot analysis of PrPres in the brains of mice: PrPres was probed with anti-PrP mAb T2. The days postinoculation (dpi) are presented on the top of the blot. NI indicates the uninfected mouse brain. The protein load in each lane is presented on the bottom of the blot. (**c**) Deposition of PrPSc stained with an SAF84 antibody in coronal sections of the brains of mBSE-inoculated mice in the cerebral cortex upper hippocampal CA1 region. CC, corpus callosum; HP, hippocampus; NC, neocortex.

To determine the amplification characteristics of mBSE prion seeding activity, we performed end-point dilution RT-QuIC on BH stocks at multiple defined time points and calculated the SD_50_ (Figure 2a). The LD_50_ of the same BH had previously been estimated using a mouse bioassay [19]. In addition, to semi-quantify the levels of PrPres, we reprocessed the same BH stocks for ELISA using the Seprion ligand [22]. Seeding doses did not increase during the first ~30 days after inoculation. However, there was a progressive increase in seeding activity (30 to 125 days) after this lag phase. A similar trend was observed for PrPres levels, whereas the infectious titer increased exponentially without any lag phase until 100 days and then reached a plateau phase. The estimated infectious titer at 100 days was 7.9 log LD_50_ per gram, which was indistinguishable from that at 140 days. These data suggested that the kinetics of infectious titer did not correlate with those of the seeding dose and PrPres levels in mBSE prion mouse models, especially in the first 30 days and from 100 to 140 days. The exponential phase of the seeding dose, infectious titer, and PrPres levels from 40 to 100 days was used to calculate each doubling time as PrPres was undetectable or barely detectable until 40 days [19], and the infectious titer reached a plateau by 100 days. The best fit exponential curve showed the relationships with seeding doses as 3,541,353* e^(0.085361*days), infectious units as 296,647* e^(0.056066* days), and PrPres (arbitrary light units) as 19.2724* e^(0.083569* days). These curves showed correlation coefficients of 0.906, 0.971, and 0.972, respectively. According to a previous study [6], the doubling times of seeding dose, infectious titer, and PrPres levels were calculated as 8.1 days, 12.4 days, and 8.3 days, respectively, from 40 to 100 days. We observed a similar doubling time of seeding activity and PrPres accumulation.

Notably, at end-stage disease, an uncoupling of seeding doses and PrPres levels was observed. The seeding doses seemed to reach a plateau level by 125 dpi, whereas PrPres levels were still elevated. A significant increase in PrPres after 125 dpi was also confirmed by western blot analysis (Figure 2b). It is conceivable that PrPSc that accumulated at late-stage disease formed larger aggregates (Figure 2c), which might have little contribution to the increase in seeding activities. To investigate this possibility, we sonicated the BHs from an mBSE-affected mouse with terminal disease and compared the seeding doses of those with or without sonication. No major differences in seeding dose were identified in comparisons of sonicated BH and untreated BH (data not shown).

### 2.3. Seeding Activity and PrPres Levels in BHs After Centrifugation

We previously reported that, after the centrifugation (20,000× *g* for 10 min) of crude brain homogenates of mBSE-affected mice, most PrPres was precipitated as a pellet (20K-ppt), whereas the supernatant (20K-sup) still contained almost similar levels of infectivity as those in crude BHs [19]. To further elucidate the relationship between seeding activity and PrPres, we performed end-point dilution RT-QuIC on the supernatant (20K-sup) and pellet (20K-ppt) obtained after the centrifugation of crude BHs from three mBSE-infected mice.

**Figure 3:**
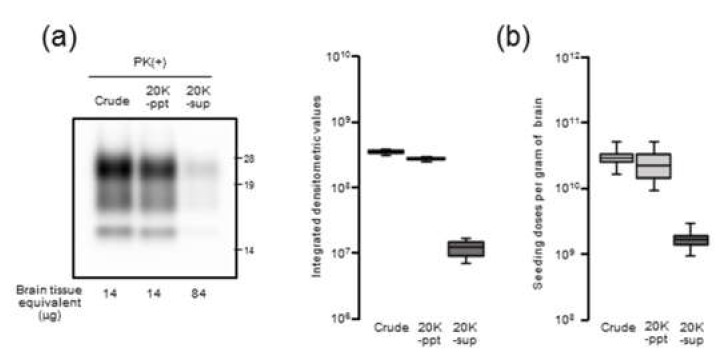
Comparison of PrPres levels and seeding activity: The 10% (w/v) brain homogenates of mouse-adopted bovine spongiform encephalopathy (mBSE)-affected mice (crude) were centrifuged at 20,000× *g* for 10 min at 4 °C. After collecting the supernatant (20K-sup), the pellet was reconstituted with the same volume of phosphate buffered saline (PBS) with brief sonication (20K-ppt). (**a**, panel at left) Western blot analysis of PrPres levels in the samples: PrPres in proteinase K (PK)-treated crude, 20K-ppt, and 20K-sup samples were detected with anti-PrP mAb T2. Brain tissue equivalents (µg) loaded per lane are indicated at the bottom of the blots. Molecular mass standards (kDa) are shown on the right side of the blots. (**a**, panel at right) Densitometric analysis of the PrPres levels: Box plots depict values from the densitometric analysis of western blots from three mBSE-inoculated mice. (**b**) RT-QuIC endpoint dilution analysis of the brain samples from three mBSE-inoculated mice. Box plots depict Spearman–Käber estimates of the SD_50_ per gram brain tissue, which was derived from three different experiments.

Semiquantitative densitometric analysis of PrPres after western blotting indicated that the 20K-sup contained ~22 and ~29-fold lower PrPres levels than the 20K-ppt and crude BHs, respectively (Figure 3a). Consistent with the western blotting results, we observed lower seeding activity for the 20K-sup (9.20 ± 0.20 log SD_50_/g) than for the 20K-ppt and crude homogenates (10.33 ± 0.32 and 10.45 ± 0.20 log SD_50_/g, respectively; Figure 3b). As the 20K-sup and crude BHs were reported to harbor similar infectivity, these results suggest potential discrepancy between RT-QuIC seeding activity and prion infectivity.

### 2.4. PK-Resistance of Seeding Activities with mBSE BHs

**Figure 4:**
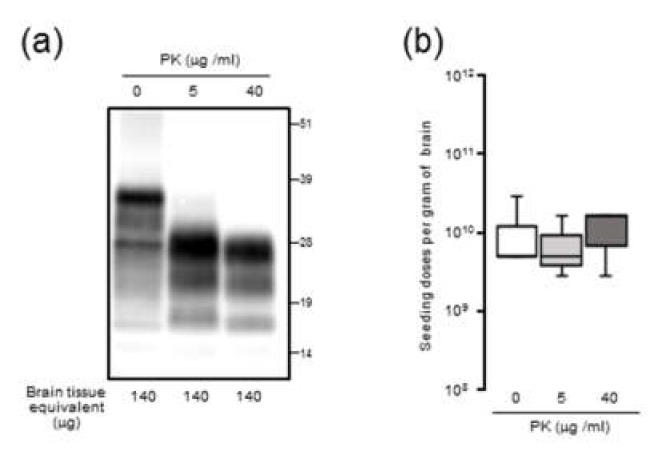
Proteinase K (PK) sensitivity of seeding activity in brain homogenates (BHs) from CD-1 mice infected with mouse-adopted bovine spongiform encephalopathy (mBSE): (**a**) Western blot analysis of PrP from mBSE samples. The mBSE BHs were pretreated with 0.5% triton X-100 (final concentration) and digested with 0, 5, and 40 µg/mL of PK. Bands were probed with anti-PrP mAb T2. (**b**) End-point dilution RT-QuIC analysis of samples: Each box represents Spearman–Käber estimates of the SD_50_ per unit per gram of brain tissue from three different experiments.

To examine the possibility that PK-sensitive PrPSc contributes to seeding activity, mBSE BHs were pretreated with triton X-100 and treated with or without PK prior to end-point dilution RT-QuIC. The PrPres was clearly detected, and intact PrPSc signal was not detected from the digested samples (Figure 4a). No significant effect of PK treatment on the mean SD_50_/g brain was seen with mBSE BH (Figure 4b).

## 3. Discussion

In the present study, we used the modified RT-QuIC method to assess prion amyloid formation and established the time course of seeding doses in the brains of mBSE-affected wild-type mice. Although the time course of seeding doses in scrapie-infected hamsters has been described [18], the temporal relationship between seeding dose and infectious titer has not yet been determined. Our temporal correlative study revealed that seeding activity correlated more tightly with a PrPres subset than prion infectivity in this model.

We observed that rinsing the 96-well plates with an acetone-ethanol mixture improved the RT-QuIC detection limit and the amyloid formation of mBSE and CWD prions. However, the precise role of organic solvents remains unclear. Possibly, the acetone-ethanol mixture might remove inhibitory factors from the surface of 96-well plates. Alternatively, a study has shown that the inhibitory effect of brain lipids on prion amyloid formation can be abrogated by alcohol-based extraction [21]. Thus, it is plausible that rinsing with the acetone-ethanol mixture might change the surface properties of 96-well plates and allows them to absorb more lipids from the samples, which would inhibit prion amyloid formation in vitro.

Our results demonstrated that there is a close relationship between prion seeding activity and PrPres levels, except for in late-stage disease, in an mBSE-affected wild-type mouse model. We followed prion seeding activity after mBSE prion infection using end-point dilution RT-QuIC. At the early stage, the lag phases were followed by an exponential phase of seeding dose as well as PrPres amplification whereas no lag phase in prion titer was observed. In the exponential phase (from 40 to 100 days), the calculated doubling time of 8.1 days for seeding dose was closer to that of 8.3 days for PrPres levels than to that of 12.4 days for infectious titer. In addition, by 100 days, the prion titer reached a plateau whereas the seeding dose and PrPres levels remained elevated. Furthermore, our RT-QuIC analysis of supernatants and pellets obtained by high-speed centrifugation of BHs suggested potential discrepancy between seeding activity and prion infectivity. These characteristics of temporal changes in seeding dose, infectious titer, and PrPres levels led us to conclude that RT-QuIC seeding activity correlates more closely with PrPres levels than with prion infectivity.

Nonetheless, at end-stage disease, an uncoupling of seeding doses and PrPres levels was observed. One possible explanation is that PrPSc that accumulated at late-stage disease might contribute little to the increase in seeding activities. Hughson and colleagues reported that a disinfectant neutralizes prion infectivity without eliminating seeding activity, implying that infectious PrPSc comprises only a subset of PrP particles with RT-QuIC activity [17]. Considering our present data together with this previous finding, it is likely that PrPSc with RT-QuIC seeding activity has a substantial but not complete overlap with proteinase-resistant PrPSc rather than infectious PrPSc.

Since it is plausible that the formation of large PrPSc aggregates at late-stage disease might affect seeding activity, BH was processed by RT-QuIC with or without sonication. Contrary to our expectations, the seeding doses of the sonicated BHs were not significantly altered compared to those of the untreated BHs, suggesting that the extent of PrPSc aggregation might exert little influence on RT-QuIC seeding activity. Although the partial fragmentation of PrPSc aggregates after the sonication of BHs was confirmed by sucrose density gradient centrifugation, we cannot exclude the possibility that our sonication treatments were insufficient to collapse the PrPSc aggregate to enhance seeding activities. Alternatively, since it is known that amyloid fibril growth begins with binding of monomers to the growing ends [23], at late-stage disease, larger aggregates of PrPSc might be mainly formed by amorphous aggregates with PK resistance which have less growing ends rather than amyloid fibrils. Including the involvement of proteinase-sensitive PrPSc and the effect of the hamster recPrP as RT-QuIC substrate, the precise mechanisms underlying an uncoupling of seeding doses and PrPres levels for PrPSc are yet to be determined.

Our present findings appear to be inconsistent with the data reported for 139A or 263K scrapie-affected Tg101LL mice, where an equivalent prion seeding dose and infectious titer but discrepant PrPres levels were observed, indicating a close correlation between prion seeding activity and infectivity [16]. One possible reason for this discrepancy might be the differences in the mice analyzed in these studies. It is plausible that, unlike PrPres in wild-type mice, only a small population of PrPres in Tg101LL mice might be responsible for seeding activity. In other words, although substantial amounts of PrPres are present in Tg 101LL mice inoculated with 139A scrapie, most of the PrPres might not be relevant for amyloid formation. Alternatively, the PrPres that accumulated in Tg101LL mice affected by 263K possibly possess higher amyloid-formation abilities than in those affected by 139A, resulting in a similar seeding activity, albeit with low amounts of detectable PrPres. To address this discrepancy, it might be necessary to conduct temporal correlative studies on seeding dose, infectious titer, and PrPres levels in Tg101LL mice affected by 139A and 263K scrapie strains.

More detailed temporal correlative studies of various prion strains are required to elucidate the relationship among prion seeding activity, infectivity, and PrPres levels. We performed a temporal correlative study with only one prion strain using wild-type mice, and thus, we cannot exclude the possibility that another prion strain in other host species might show a different correlative relationship between seeding dose and infectious titer. Indeed, in a scrapie-affected hamster model where a continuous increase in both PrPSc levels and infectious titer to the end of disease is observed [24], the seeding dose also increased toward the end of the incubation period with no plateau phase [18].

To the best of our knowledge, we believe that this is the first correlative study on the kinetics of prion seeding dose, infectious titer, and PrPres levels in parallel. We demonstrated that amyloidogenic PrPSc substantially but not completely overlaps with PrPres, as compared to infectious PrPSc, in this model. Further studies will be required to clarify the relationship among different types of PrPSc based on various prion strains in another host species.

## 4. Materials and Methods

### 4.1. Mouse-Adapted Classical BSE Brain Homogenates

Mouse-adapted classical BSE prions were propagated first in RIII mice and then sequentially in 3-week-old specific pathogen-free female CD-1 mice 14 times. In our previous study [19], 20 µL of mBSE brain homogenates (BHs), which contained 10^5.9^ LD_50_ of mBSE prion, was intracerebrally inoculated into CD-1 mice. Three recipient mice were sacrificed at designated time points (5, 30, 40, 60, 70, 90, 100, 125, and 140 days postinfection), and the brains were collected. The brains at each time point were homogenized in phosphate buffered saline (PBS) and stored at −80 °C until further use. In the current study, we used these BHs for the kinetic analysis of seeding dose, infectious titer, and PrPres levels. The data regarding infectious titers which were estimated by incubation-time assay using four to six CD-1 mice were obtained from our previous report [19] with the permission of its authors including the corresponding author.

### 4.2. Recombinant Prion Protein

Recombinant Syrian hamster prion protein (recPrP; amino acids 90–231) constructs were expressed in *Escherichia coli* strain BL21-CodonPlus (DE3)-RIPL (Stratagene, La Jolla, CA, USA) and purified as previously described with slight modifications [25]. The solubilized inclusion body containing recPrP was applied to an Ni-NTA Superflow resin (Qiagen, Venlo, Netherland) and loaded onto a XK16-20 column (GE Healthcare, Buckinghamshire, England) connected to an AKTA_FPLC_ system. The elution peak was pooled and dialyzed in dialysis buffer containing 100 mM NaCl in 10 mM NaPO_4_, pH 5.5, overnight at 4 °C. Concentrations of recPrP were estimated by its absorbance at 280 nm. Aliquots of proteins were stored at −80 °C until further use. After thawing, aliquots of proteins were centrifuged at 20,000× *g* for 30 min at 4 °C and the soluble recPrP was used.

### 4.3. Rinsing of 96-Well Plate

Optical-bottom black 96-well plates (#265301, Thermo Fisher Scientific, Waltham, MA, USA) were rinsed with an acetone-ethanol 1/9 (v/v) mixture. Each well was filled with 320 μL of acetone–ethanol mixture and aspirated completely. The rinsed 96-well plate was dried in a safety cabinet for at least 15 min before use.

### 4.4. Real-Time QUIC

Real-time QuIC (RT-QuIC) assays were performed as reported previously with slight modifications [25]. Briefly, 98 µL of the RT-QuIC reaction substrate mixture containing 0.1 mg/mL Syrian hamster recPrP (aa 90–231), 320 mM NaCl, 1 mM ethylenediaminetetraacetic acid (EDTA), and 10 µM thioflavin T (Th T) in 100 mM phosphate (pH 7.4) was loaded into an optical-bottom black 96-well plate. The reaction substrate mixtures were seeded with 2 μL of BH 10-fold serially diluted with 0.075% sodium dodecyl sulfate (SDS) in PBS. The seeded brain tissue equivalents from an mBSE-inoculated mouse were diluted to 2 µg to 200 fg (10^−2^ to 10^−9^ dilutions of 10% weight per volume of the BH), and those from a normal mouse were diluted to 20 ng. Each brain homogenate was assayed in quadruplicate wells at least three times using independent reaction substrate mixtures. The plates were sealed and incubated in a BMG FLUOstar Omega plate reader (BMG LABTECH, Ortenberg, Germany) at 37 °C with 1 min of shaking (1100 r.p.m., double-orbital) and 1 min of rest for 62 h. ThT fluorescent readings (450-nm excitation and 480-nm emission, bottom-read, 20 flashes per well, gain of 1500) were recorded every 15 min. The threshold for positivity of each BH was calculated as four-fold greater than the average of the initial fluorescence of all samples. The lag phase (h) was defined as the reaction time required to exceed the threshold. Amyloid formation rate (AFR; 1/h), which is the inverse of lag phase, was used as an indicator of amyloid seeding activity. RT-QuIC conditions such as the concentration of SDS, reaction temperature, and shaking speed were optimized to minimize the false positive rate without a loss of sensitivity.

### 4.5. Calculation of Seeding Dose

Seeding doses of 50% (SD_50_) were determined using end-point dilution RT-QuIC. The log-dose of the dilution yielding 50% positive replicates per gram wet weight equivalent (indicated as log SD_50_ per gram) was calculated using the Spearman–Kärber method, with 10-fold dilutions of each of the positive and negative seeding activity ratios.

### 4.6. Enzyme-Linked Immunosorbent Assay (ELISA)

The BHs were processed for ELISA using the Seprion ligand (Microsens Biotechnologies, London, UK) to semi-quantify the levels of PrPres [22]. The Seprion ligand is a group of polymeric compounds with specificity for PrPSc and can be immobilized directly on the wells of a microplate as a substitute for capturing antibodies. Briefly, the BHs were lysed in capture buffer (1% N-lauroyl sarcosine, 1% triton X-100, 1% bovine serum albumin, and 0.5% trypsin in 50 mM tris–HCl, pH 8.3) and applied to a 96-well microplate (FluorNunc Black, Thermo Fisher Scientific) coated with the Seprion ligand. After incubation for 2 h, PrPres captured on the well was denatured with 4 M guanidine thiocyanate in 20% polyethylene glycol 8000 (Fisher Scientific, Hampton, NH, USA) and detected using anti-PrP mAb T2 conjugated with horse radish peroxidase (HRP) [26]. The plates were developed using a chemiluminescence solution (Supersignal West Dura Extended Duration Substrate; Pierce Biotechnology, Rockford, IL, USA), and the signal was determined using an ARVO SX 1420 multilabel counter (Wallac, Turku, Finland). Four-fold serial dilutions of the BH from a terminally ill mouse with mBSE were subjected to ELISA to confirm its linear dynamic range (Figure S1).

### 4.7. Western Blot Analysis

The 10% (w/v) BHs of mBSE-affected mice were centrifuged at 20,000× *g* for 10 min at 4 °C. After collecting the supernatant (20K-sup), the pellet was reconstituted with the same volume of PBS with brief sonication (20K-ppt). Each sample was diluted 20-fold with lysis buffer containing 10 mM tris-HCl (pH 7.4), 150 mM NaCl, 1 mM EDTA, 0.5% triton X-100, and 0.5% sodium deoxycholate. For PrPres detection, each sample was treated with 40 µg proteinase K (PK) for 30 min at 37 °C. For proteinase-sensitive PrPSc analyses, the 10% (w/v) BHs of mBSE-affected mice were pretreated with 0.5% triton X-100 (final concentration) and digested with 0, 5, and 40 µg/mL of PK for 30 min at 37 °C. PK digestion was terminated by adding Pefabloc (Roche Diagnostics, Mannheim, Germany) at a final concentration of 4 mM. All samples were suspended in three times their volumes of 4 × lithium dodecyl sulfate sample loading buffer (Thermo Fisher Scientific) and boiled. The samples were electrophoresed on 12% NOVEX pre-cast gels (Thermo Fisher Scientific) and electrotransferred onto Durapore (Merck Millipore, Burlington, MA, USA) polyvinylidene fluoride membranes. The blots were probed with HRP-conjugated PrP-specific antibody T2. Chemilumi One Super (Nakarai Chemical, Kyoto, Japan) was used for immunodetection. The blots were visualized with a Fluorchem (Alpha Innotech, San Leandro, CA, USA) and analyzed using image reader software (AlphaEaseFC; Alpha Innotech) according to the manufacturer’s instructions.

### 4.8. Immunohistochemistry

Formalin-fixed left hemispheres were embedded in paraffin wax following immersion in 98% formic acid to decrease infectivity. After epitope retrieval, PrPSc immunocytochemistry was performed using the anti-PrP monoclonal antibody SAF84. As the secondary antibody, the anti-mouse universal immunoperoxidase polymer (Nichirei Histofine Simple Stain MAX-PO (M); Nichirei, Tokyo, Japan) was used. PrPSc was visualized with 3,3′-diaminobenzedine tetrachloride as the chromogen.

### 4.9. Statistical Analysis

Values in the text are expressed as means ± SD. To determine statistical significance, a Student’s *t* test for paired samples was used. Statistical analysis of the data was performed using Excel and R software programs.

## Supplementary Materials

The following are available online at https://www.mdpi.com/2076-0817/9/3/241/s1, Figure S1: Detection of PrPres accumulation in the brain of mouse-adopted bovine spongiform encephalopathy (mBSE)-affected mice using ELISA with the Seprion ligand, Figure S2: RT-QuIC detection of prion seeding activity in the brains of elk affected by chronic wasting disease.
