# Supplementary material for: PrPSc with Seeding Activity Extensively Overlaps with Proteinase-Resistant PrPSc Rather than Infectious PrPSc
Source: Pathogens. 2020 Mar 24;9(3):241. doi: 10.3390/pathogens9030241 (PMC7157578; doi:10.3390/pathogens9030241)
Supplement: Supplementary file 1 [file pathogens-09-00241-s001.pdf]

Supplementary Materials

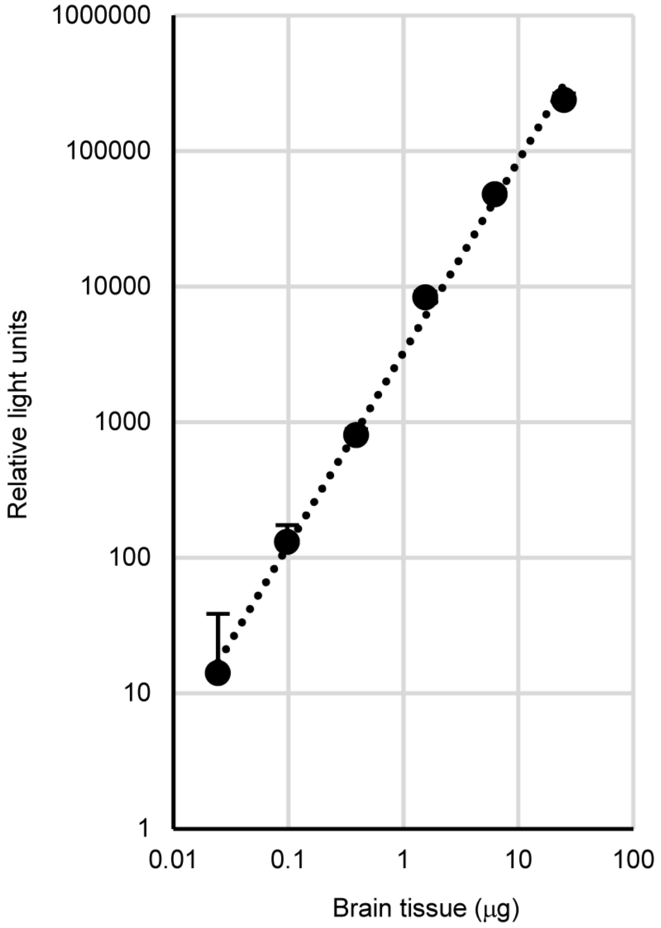

Figure S1. Detection of PrPres accumulated in the brain of mBSE-affected mouse using ELISA with the Seprion ligand. Four-fold serial dilutions of 10% (w/v) brain homogenates of a terminally ill mBSE-affected mouse were examined using ELISA with the Seprion ligand. Representative ELISA data are shown. Each point represents the mean ± SD and derived from three replicates (filled black circle). A linear relationship was observed (dashed line: y = 3245.2* x^ (1.14163) R^2 = 0.9967, where y is relative light units and x is amount of the brain tissue).

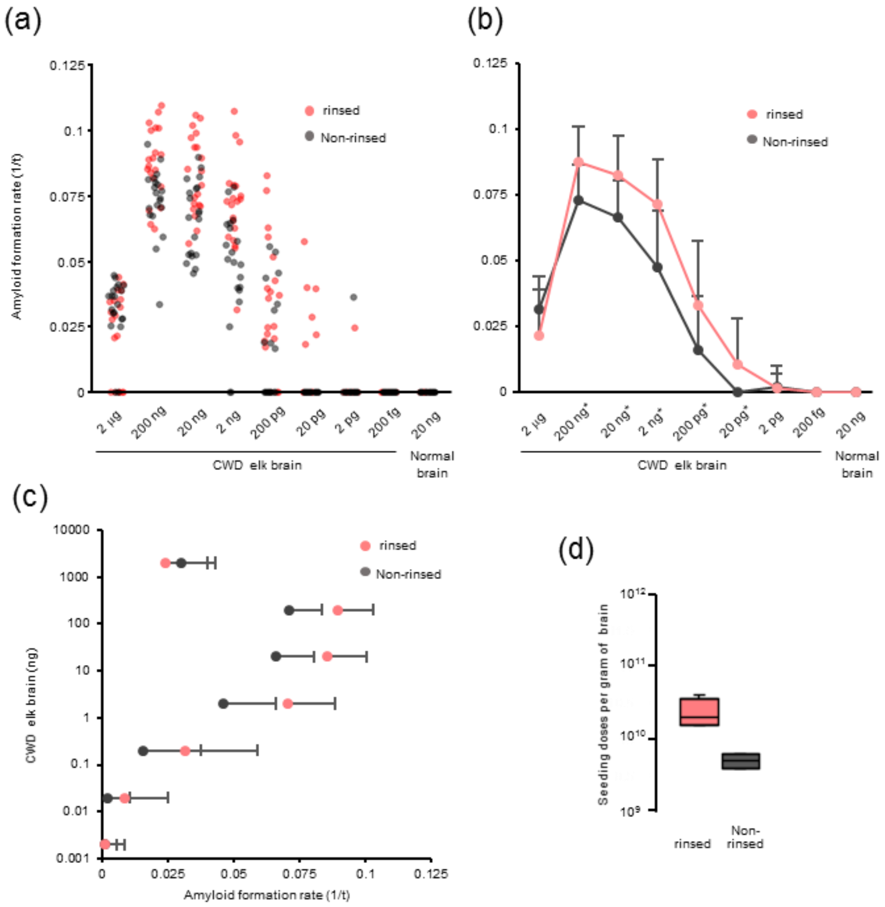

Figure S2. Real-time quaking-induced conversion (RT-QuIC) detection of prion seeding activity in the brain from elk affected with chronic wasting disease (CWD). Syrian hamster recombinant prion protein (amino acids 90–231) was used as a substrate for RT-QuIC reactions. RT-QuIC reaction mixtures were seeded with 2 µg to 200 fg brain tissues (10^{-2} to 10^{-9} dilutions of 10% weight per volume brain tissues) from CWD elk or with 20 ng brain tissue from a normal deer (NBH). Data are derived from quintuplicate wells of four experiments for each brain tissue dilution. (**a**) Each dot represents the amyloid formation rate (AFR) measured using a 96-well plate rinsed with (pale red) or without (grey) acetone-ethanol mixture. (**b**) Line plot of (a). The mean ± SD AFR is displayed for each dilution. Significant differences at $p < 0.05$ (*) between AFR measured using rinsed and non-rinsed 96-well plate are indicated. **(c)** Plot of the data sets of (b) on a single set of graphs without NBH. (**d**) Each box represents Spearman–Käber estimates of the SD_{50} per unit per gram of brain tissue measured in a 96-well plate rinsed with (pale red) or without (grey) an acetone-ethanol mixture. Data are derived from quintuplicate wells from four experiments for each brain tissue dilution.
